# Economic burden of varicella complications in two referral centers in Mexico

**DOI:** 10.1080/21645515.2018.1504541

**Published:** 2018-10-23

**Authors:** Mercedes Macias-Parra, Miguel Angel Rodriguez-Weber, Sarbelio Moreno-Espinosa, Berenice Ceron-Trujillo, Karla Ojeda-Diezbarroso, Rodrigo DeAntonio, Ricardo Cortes-Alcala, Gustavo Martinez, Roberto Carreño-Manjarrez, Rodolfo Norberto Jiménez-Juárez

**Affiliations:** a Instituto Nacional de Pediatría, Ciudad de México, México; b Departamento de Infectología, Hospital Infantil de México Federico Gómez, México City, México; c GSK, Panamá City, Panamá; d GSK, México City, México

**Keywords:** Complications, cost, Mexico, varicella, vaccination

## Abstract

Varicella-zoster virus causes varicella (chicken-pox), mainly in young children. Most cases are mild but serious complications can occur, resulting in significant morbidity and mortality. The objective of this study was to estimate the cost burden of varicella hospitalizations in two pediatric reference hospitals in Mexico. This retrospective observational study collected data on patients aged <18 years admitted to two third-level referral hospitals in Mexico. Cases were identified from hospital records using International Classification of Diseases Ninth Revision (ICD-9) codes 052 Chickenpox, or Tenth Revision (ICD-10) codes B01 Varicella (chickenpox). Data on demographic and clinical characteristics and resource use were collected from hospital records. Costs for hospital stay and interventions were obtained from the Mexican Institute for Social Security for 2015 and updated to 2017 costs. A total of 172 hospitalized varicella clinically-confirmed cases and 121 varicella- contacts (with epidemiological linkage to a clinically-confirmed case) were included. Thirty eight of the 172 cases (22.0%) experienced complications. There were no deaths. The median duration of hospitalization was 12 days for cases and 23 days for contacts. The median hospitalization cost was MXN 82,572 (USD 4,434) per case, and MXN 89,453 (USD 4,804) per contact. Although considered a mild disease, varicella was associated with a substantial cost burden in two Mexican third-level referral hospitals.

## Introduction

Varicella-zoster virus causes varicella (chicken-pox), which is characterized by a vesicular rash and mainly occurs in young children.^1^ After the chicken-pox has resolved, the virus can remain latent in dorsal root spinal ganglia. When reactivated later, which can occur at any time of life although it is more frequent at older ages, the virus causes herpes zoster (shingles).^2^ Varicella is highly infectious, and secondary cases occurs in 61–100% of susceptible household contacts.^3^ Varicella infection is usually mild to moderate in individuals with normal immune function, although even an uncomplicated severe case can result in over 1,000 lesions and severe symptoms.^1^ Serious complications of varicella requiring hospitalization can occur,^4^ including secondary bacterial infections, central nervous system involvement, pneumonia, and skin and soft tissue infections.^1,2^ Complications can be severe or fatal, even in healthy individuals.^3,5,6^

Varicella is associated with a substantial burden of morbidity and mortality. A total of 62,246 hospitalizations associated with varicella zoster virus were recorded between 2008 and 2013 in Brazil before the introduction of universal vaccination with the tetravalent measles-mumps-rubella-varicella vaccine.^2^ The majority of these varicella-related hospitalizations occurred in children aged < 9 years.^2^ The incidence of hospitalized varicella in children aged < 15 years was 15.7 per 100,000 per year in Australia prior to the 1-dose varicella mass vaccination,^7^ and the estimated incidence of varicella-related hospitalization was 5.29–6.89 per 100,000 in children aged 0–15 years in Turkey.^4^ Varicella mortality rates in Brazil averaged 0.88 per 100,000 per year in infants aged < 1 year and 0.4 per 100,000 in children aged 1–4 years^2^. In a study in Colombia, 13 of 513 (2.5%) children hospitalized for varicella complications died.^5^

Information on the cost and burden of varicella complications will be important for the economic evaluation of preventive strategies such as immunization. A few studies have investigated the cost of varicella hospitalizations in Colombia,^8^ Panama,^5^ Chile,^6^ and Turkey.^9^ However, there is currently no information available from hospitals in Mexico on the cost of varicella-associated hospitalizations.

The objective of this retrospective observational study was to fill this gap in knowledge by investigating the clinical and cost burden of varicella hospitalizations in individuals aged < 18 years admitted to two third-level referral pediatric hospitals in Mexico.

## Results

A total of 172 hospitalized varicella-confirmed cases and 121 varicella contacts were included in the study across the two participating hospitals. In the Hospital Infantil de Mexico “Federico Gomez” (HIMFG), only varicella cases were reported, while the Institituto Nacional de Pediatria (INP) reported also all the contacts who had exposure to a clinically-confirmed case during hospitalization.

Table 1 summarizes the demographic and clinical characteristics of the subjects across both hospitals. Only 2 cases and no contacts were reported in 2011; the other three study years had broadly comparable number of cases and contacts in each year. In 154 cases (89.5%), comorbidities were reported, meanwhile, 107 contacts (88.4%) reported any comorbidity.

**Table 1.** Demographic and clinical characteristics of study population.

Parameter	Cases (n = 172)	Contacts (n = 121)	Total (n = 294)
**Participating hospital**			
HIMFG	100	-	100
INP	72	121	194
**Sex**			
Females, n (%)	73 (42.4%)	53 (43.8%)	126 (43.0%)
Males, n (%)	99 (57.6%)	68 (56.2%)	167 (57.0%)
**Age group**			
< 1 year, n (%)	10 (5.8%)	45 (37.2%)	55 (18.8%)
1–4 years, n (%)	68 (39.5%)	40 (33.1%)	108 (36.9%)
5–9 years, n (%)	58 (33.7%)	19 (15.7%)	77 (26.3%)
10–17 years, n (%)	36 (20.9%)	17 (14.1%)	53 (18.1%)
**Source of contagious contact**			
Outside hospital, n (%)	52 (71%)	5 (4%)	57 (29%)
In hospital, n (%)	21 (29%)	116 (96%)	137 (71%)
**Year**			
2011, n (%)	2 (1.2%)	0	2 (0.7%)
2012, n (%)	57 (33.1%)	43 (33.5%)	100 (34.1%)
2013, n (%)	54 (31.4%)	37 (30.6%)	91 (31.1%)
2014, n (%)	59 (34.3%)	41 (33.9%)	100 (34.1%)
**Other**			
Previous varicella vaccination, n (%)	3 (1.7%)	5 (4.1%)	8 (2.7%)
Presence of co-morbidities, n (%)	154 (89.5%)	107 (88.4%)	261 (89.1%)

HIMFG, Hospital Infantil de México Federico Gomez; INP, Instituto Nacional de Pediatría, n, number of cases

Thirty eight of the 172 varicella-confirmed cases (22.1%) experienced complications. Among them, 23 occurred in varicella cases with comorbidities (60.5%). None of the contacts developed varicella symptoms or any complications (Table 2). The most common complications were sepsis, septic shock and pneumonia (Table 2). There were no varicella-associated deaths. All except five cases were discharged without sequelae. Of these five cases, one subject had functional limitation in one extremity and scars, and the other four had skin sequelae associated with scars. All were aged 1–10 years.

**Table 2.** Complications of varicella cases.

Complications	Cases N = 172	Contacts N = 121	Total N = 293
None, n (%)	134 (77.9%)	121 (100%)	255 (87.6%)
Any complication, n (%)	38 (22.1%)	-	38 (13.4%)
Severe sepsis and septic shock, n	15	0	15
Low respiratory tract, n	8	0	8
Skin and soft tissue, n	7	0	7
Neurological, n	5	0	5
Hemorrhagic varicella, n	1	0	1
Nosocomial sepsis, n	1	0	1
Bacterial superinfection, n	1	0	1

n, number of cases

**Figure 1.**
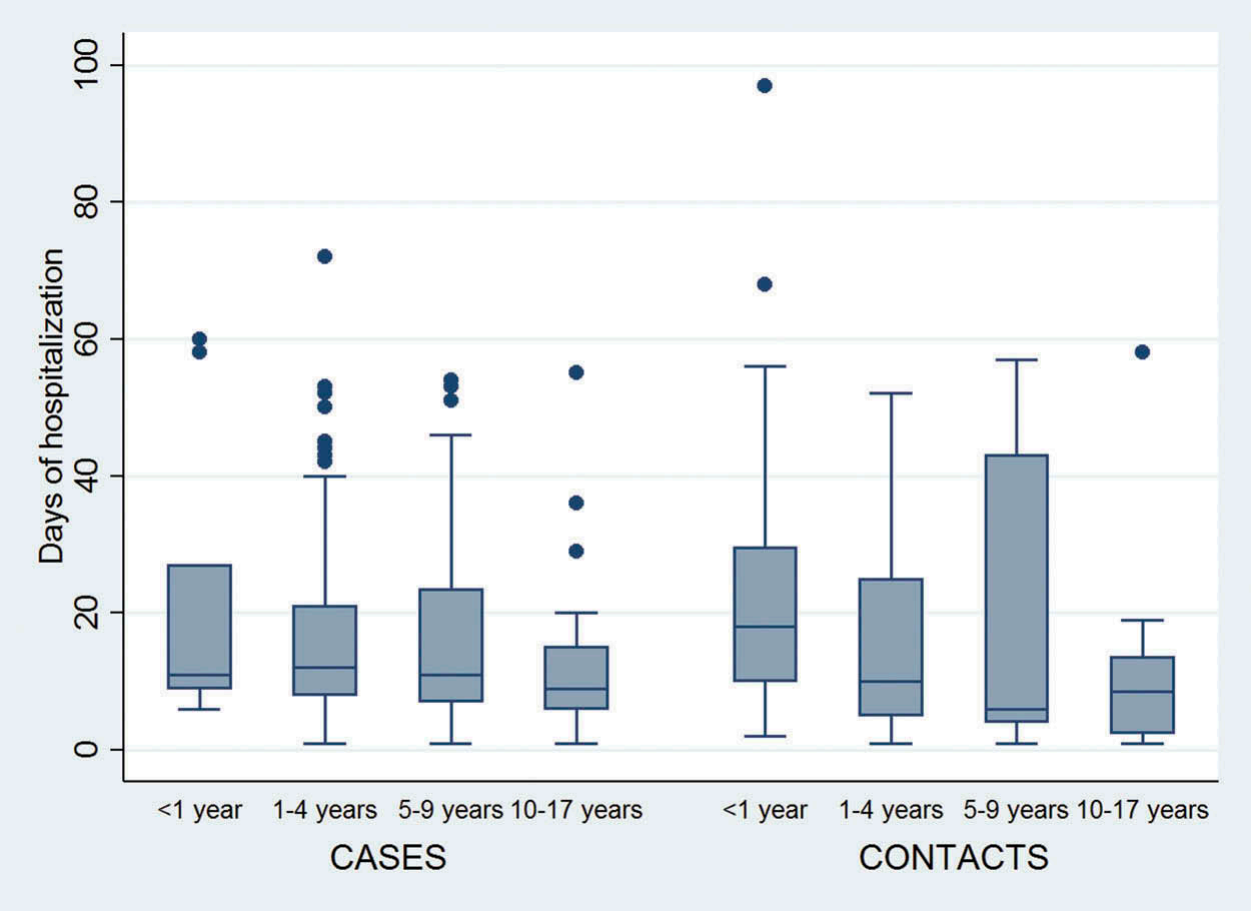
Duration of Hospital Stay for Varicella-Confirmed Cases and Varicella Contacts By Age Group. ***Box:*** *median with 25th and 75th percentiles; **Whiskers**: upper/lower adjacent values; **Data points**: outside values.*

Figure 1 shows the duration of hospitalization by age group for varicella-confirmed cases and varicella contacts. The median duration of hospitalization was 26.2 days (standard deviation [SD] 133.1) for varicella-confirmed cases, and 13 days (SD 17.3) for varicella contacts. (Table 3)

**Table 3.** Hospital resource use and cost for varicella hospitalizations.

	Cases (n = 172)	Contacts (n = 121)	Estimated cost per day/unit, MXN
Median hospitalization, days (SD; percentile 10th – 90th)	12 (133.1; 5 – 53)	13 (17.3; 3 – 44)	6881
Antivirals, % (n)	89.0% (n = 153)	64.5% (n = 78)	403
Intravenous	82.6% (n = 142)	43.0% (n = 52)	415
Oral	19.8% (n = 34)	29.8% (n = 36)	10
Mean days (SD; range)	8.4 (4.1; 1–20)	6.1 (3.3; 1–19)	
Immunoglobulin intravenous, % (n)	2.3% (n = 4)	0	6690
Antibiotics, % (n)	34.9% (n = 60)	0	
Serology, % (n)	1.4% (n = 1)	0	920
X-rays, % (n)	26.9% (n = 46)	0	441
Cultures, % (n)	30.2% (n = 52)	0	755
Intensive care unit, % (n)	12.8% (n = 22)	0	33,911

MXN, Mexican peso; SD, standard deviation; n, number of cases

Table 3 shows hospital resource use and cost for varicella-confirmed cases and varicella contacts. No differences were observed between hospitals. Antiviral treatment was given in 89% of varicella-confirmed cases and 64.5% of varicella contacts, administered as prophylaxis. The median days of treatment with acyclovir for contacts was 7 days. They did not develop varicella.

The estimated median hospitalization cost was MXN 82,572 (USD 4,434) per varicella-confirmed case ranging from MXN 34,405 to MXN 364,693 (USD 1,847 – 19,586), and MXN 89,453 (USD 4,804) per varicella contact, ranging from MXN 20,643 to MXN 302,764 (USD 1,109 – 16,260). Nonetheless, for contacts the median cost attributed to varicella antiviral treatment was MXN 48,167 (USD 542).

For varicella-confirmed cases with any comorbidity, the median hospitalization cost was MXN 75,680 (USD 4,064) corresponding to a median hospitalization of 11 days, meanwhile, in cases without comorbidities, the median duration of hospitalization was 20 days representing MXN 137,600 (USD 7,390).

## Discussion

This retrospective observational study provides the first published data from Mexico on the cost of varicella-related hospitalizations, using information derived from individuals aged < 18 years admitted to two third-level referral hospitals. The median hospitalization cost was MXN 82,572 (USD 4,434) per varicella-confirmed case, and MXN 89,453 (USD 4,804) per varicella contact. Both hospitals are comparable in resources use and cost since they belong to the Coordination of National Institutes of Health, and also are comparable in the attended population. It should be noted that both hospitals in our study are third-level referral hospitals, which are likely to receive particularly complicated cases or patients with significant co-morbidities requiring specialist management, and so these costs may not be representative of the costs incurred in routine hospital care in Mexico. Nevertheless, our results indicate that although varicella is commonly considered a mild disease, varicella-related hospitalizations in Mexico can be associated with substantial costs.

The median length of stay reported in this study was 14 days for varicella-confirmed cases and 13 days for varicella contacts. These are longer than the length of stay reported by previous studies in Panama (median length of stay 8.9 days),^5^ Turkey (median length of stay 6 days,^4^ and Thailand (median duration 6 days).^10^ The median cost of hospitalization in the current study was also higher than the study in Panama (USD1,209),^5^ which would be consistent with the longer duration of hospitalization. As noted above, both hospitals in our study were third-level referral hospitals that might be expected to admit more complicated patients than the average hospital, and this may explain the longer length of stay compared with other published studies. Our results represent two of the main pediatric reference hospitals in Mexico, which might limit the extrapolation of the findings to other centers, however the complications reported in patients with varicella are similar to those reported by other authors, and the most important aspect is that varicella complication may be very severe not only life threatening but also with long term sequelae and the disease can be prevented with vaccination. Other mild or moderate features requiring less complex attention in primary or secondary facilities might not be represented in our report.Most of the varicella-confirmed cases in our study had a source of contact outside the hospital, but a significant number of intrahospital contacts had to receive antiviral prophylaxis and hospitalization had to be extended due to varicella exposure. This finding may have implications for health service management, as it implies a considerable risk of varicella transmission to other patients within the hospital setting. A patient hospitalized with varicella may, therefore, result in additional nosocomial varicella cases occurring within the hospital. This in turn could potentially place a strain on hospital resources, or require closing beds in the hospital wards, intensive care unit and use of antiviral prophylaxis to limit the spread of infection. Clearly, these could have adverse effects on patient care. We have not attempted to quantify the potential impact of such consequences in this study.

Our retrospective study design has some limitations. International Classification of Diseases (ICD) codes may be missing or incorrectly entered in the hospital records, which may result in some varicella patients being missed from the study. Data on resource use may be missing or incomplete at the time of data collection. Some costs and resource use in patients with co-morbidities or with other conditions (e.g. in patients who contracted nosocomial varicella infections whilst hospitalized for other reasons) may relate to conditions other than varicella., nonetheless, hospitalization costs were higher in varicella cases of subjects without comorbidities, mostly related to a longer duration of hospital stay. The retrospective analysis did not allow determining which interventions and costs may or may not be attributed to varicella itself or to other conditions..

## Conclusion

Although conventionally considered a mild disease, varicella in children and adolescents aged < 18 years was associated with a substantial cost burden in two Mexican third-level referral hospitals. Furthermore, the high proportion of varicella contacts with an in-hospital source of varicella indicates a high risk of varicella transmission to other patients in a hospital, with consequent potential for placing strain on hospital resources or requiring closure of beds for infection control. Preventive interventions such as vaccination have the potential to reduce this burden of varicella illness in hospitals. The substantial cost of varicella hospitalizations should be taken into account in economic evaluations of preventive strategies.

## Methods

This retrospective observational study was conducted at two third-level referral hospitals in Mexico, HIMFG and INP. Both hospitals have highly specialized staff and technical equipment, clinical services highly differentiated by function and conduct teaching activities, as per World Health Organization definition.^10^

### Study population

All varicella-related hospitalizations, including healthcare-associated infections, occurring at the two participating hospitals in children aged < 18 years during the period 2011–2014 were identified from the hospital records. Cases were identified from hospital records through the epidemiological unit or department of statistics, using codes from the ICD Ninth or Tenth Revision (ICD-9 or ICD-10), as indicated in Table 4.

**Table 4.** Varicella international classification of diseases ninth or tenth revision (ICD-9 or ICD-10).

ICD-9 codes	ICD-10 codes
052 Chickenpox;	B01 Varicella (chickenpox);
052.0 Postvaricella encephalitis;	B01.0 Varicella meningitis;
052.1 Varicella (hemorrhagic) pneumonitis;	B01.1 Varicella encephalitis, myelitis and encephalomyelitis;
052.2 Postvaricella myelitis; 052.7 Chickenpox with other specified complications convert;	B01.11 Varicella encephalitis and encephalomyelitis;
052.8 Chickenpox with unspecified complication;	B01.12 Varicella myelitis;
052.9 Varicella without mention of complication.	B01.2 Varicella pneumonia; B01.8 Varicella with other complications; B01.81 Varicella keratitis;
	B01.89 Other varicella complications; B01.9 Varicella without complication.

To maximize the sensitivity of the search, admission and discharge diagnoses were used as references. Diagnosis at discharge was the main criterion for inclusion because it indicated a valid confirmation of varicella-related hospitalization and complications.

The study enrolled previously healthy children as well as children with immunosuppression or chronic conditions.

A varicella-clinically-confirmed case was defined as an illness with acute onset of diffuse (generalized) maculo papulo vesicular rash without other apparent cause, with or without laboratory confirmation and previous epidemiological linkage to a varicella-case. Contacts were defined as the exposure to a varicella clinically confirmed case during hospitalization.

### Data collection

Data on admission time, gender, age, receipt of varicella vaccine, underlying conditions, reason for admission, nature and type of any varicella-associated complications, and presence of varicella serology were collected from the hospital records.

Resource use data including the length of hospital stay, intensive care unit stay, use of mechanical ventilation, use of radiological techniques, blood culture results, administration of acyclovir (dose and duration if available), administration of intravenous immunoglobulin (dose and duration if available), antibiotic therapy, and hospitalization outcome (survival to hospital discharge, sequelae at hospital discharge, or death) were collected by review of medical charts.

For contacts, the length of hospital stay was estimated using as reference the date when occurred the exposure to a varicella clinically confirmed case during hospitalization. The number of days of antiviral administration were also estimated for these subjects.

Costs for hospital stay and interventions were obtained from the Mexican Institute for Social Security for 2015,^11^ and updated to 2017 costs using the consumer price index published by the Mexican National Institute of Statistics and Geography.^11^ All costs were calculated in Mexican pesos (MXN). For ease of comparison with other published studies, data were also converted to USD using an exchange rate of 1 USD = 18.62 MXN.^12^

The study was conducted in accordance with all applicable regulatory requirements, including all applicable subject privacy requirements and the guiding principles of the Declaration of Helsinki. Approval by the local or institutional committee was obtained before any data extraction, and data privacy was protected by using anonymized data.

### Statistical analysis

Demographic characteristics and healthcare resource use data were summarized using descriptive statistics, including frequency tables for categorical data and mean, median, range, 95% confidence intervals (CI), SD and/or standard error for continuous variables. For cost analyses, median cost was calculated stratified by subjects with and without underlying disease.

## Abbreviations

CI: confidence interval
HIMFG: Hospital Infantil de Mexico “Federico Gomez”
ICD: International Classification of Diseases
INP: Instituto Nacional de Pediatria
MXN: Mexican peso
SD: standard deviation
USD: US Dollar

## References

[1] Heininger U, Seward JF. 2006 Varicella. Lancet. 368:1365–1376. doi:10.1016/S0140-6736(06)69561-5. 17046469

[2] De Martino Mota A, Carvalho-Costa FA 2016 Varicella zoster virus related deaths and hospitalizations before the introduction of universal vaccination with the tetraviral vaccine. J Pediatr (Rio J). 92:361–366. doi:10.1016/j.jped.2015.10.003. 26969400

[3] Lopez A, Schmid S, Bialek S Varicella; 2011 [2017 Accessed 2017 Sept 30]. http://www.cdc.gov/vaccines/pubs/surv-manual/chpt17-varicella.html.

[4] Dinleyici EC, Kurugol Z, Turel O, Hatipoglu N, Devrim I, Agin H, Gunay I, Yasa O, Erguven M, Bayram N, et al The epidemiology and economic impact of varicella-related hospitalizations in Turkey from 2008 to 2010: a nationwide survey during the pre-vaccine era (VARICOMP study). Eur J Pediatr. 2012;171:817–825. doi:10.1007/s00431-011-1650-z. 22170238

[5] Saez-Llorens X, De Suman O, De Moros D, Rubio Mdel P Complications and costs associated with chickenpox in immunocompetent children. Rev Panam Salud Publica. 12;2002:111–116. 12243696 10.1590/s1020-49892002000800006

[6] Abarca K, Hirsch T, Potin M, Perret C, Zamorano J, Gonzalez C, Vial P [Complications in children with varicella in 4 hospitals in Santiago, Chile: clinical spectrum and estimation of direct costs]. Rev Med Chil. 129;2001:397–404. 11413992

[7] Carapetis JR, Russell DM, Curtis N 2004 The burden and cost of hospitalised varicella and zoster in Australian children. Vaccine. 23:755–761. doi:10.1016/j.vaccine.2004.07.025. 15542199

[8] Alvis-Guzman N, Paternina-Caicedo A, Alvis-Estrada L, De La Hoz-Restrepo F Direct costs of complicated chicken pox in a Colombian pediatric population. Rev Salud Publica (Bogota). 13;2011:921–929. 22634994 10.1590/s0124-00642011000600005

[9] Turel O, Bakir M, Gonen I, Hatipoglu N, Aydogmus C, Hosaf E Children hospitalized for varicella: complications and cost burden. Value Health Reg Issues. 2013:226–230. doi:10.1016/j.vhri.2013.05.003. 29702869

[10] World Health Organization Management of health facilities: referral systems; 2017 Accessed 2017 Sept 30 http://www.who.int/management/facility/referral/en/index2.html.

[11] Diario Oficial de la Federación Costos Unitarios por Nivel de Atención Médica para el ejercicio 2015; 2015 2017 Accessed 2017 Sept 30 http://www.dof.gob.mx/nota_detalle.php?codigo=5381602&fecha=11/02/2015.

[12] Secretaria de Gobernacion Diario Oficial de la Federacion Tipo de cambio y Tasas; 2017 Accessed 2017 9 30 http://dof.gob.mx/indicadores.php.

